# Comparisons of Waist Circumference Measurements at Five Different Anatomical Sites in Chinese Children

**DOI:** 10.1155/2017/7678613

**Published:** 2017-02-02

**Authors:** Chaoran Yang, Lin Wang

**Affiliations:** ^1^Department of Sports Rehabilitation, Shanghai University of Sport, Shanghai, China; ^2^Key Laboratory of Exercise and Health Science of the Ministry of Education, Shanghai University of Sport, Shanghai, China

## Abstract

This study compared the waist circumference (WC) measurements of Chinese children at different sites to determine the relationship between WC measurements and body fat. WC was measured at five sites in 255 subjects aged 9–19 years: immediately below the lowest rib (WC1), at the narrowest waist (WC2), the midpoint between the lowest rib and the iliac crest (WC3), 1 cm above the umbilicus (WC4), and immediately above the iliac crest (WC5). Body fat mass (FM), body fat percentage (% BF), body fat mass in the trunk (FM in the trunk), and fat percentage in the trunk (% BF in the trunk) were determined by dual-energy X-ray absorptiometry. The WCs were then compared through ANOVA with repeated measurement. The relationship of WC of each site with FM, % BF, FM in the trunk, and % BF in the trunk was examined through partial correlation. The WCs exhibited the following pattern: WC2 < WC1 < WC3 < WC4 < WC5 (*p* < 0.001) in males and WC2 < WC1 < WC4, WC3 < WC5 (*p* < 0.001) in females. The measured WCs were strongly correlated with FM, % BF, FM in the trunk, and % BF in the trunk. The WC measurements at five commonly used sites among Chinese children are different from one another. Results indicate that standardizing the anatomic point for the WC measurements is necessary.

## 1. Introduction

Childhood obesity is a critical health challenge in the 21st century [1]. Childhood obesity is significantly associated with the cardiovascular risk factors among children and adolescents [2]. Other childhood clinical consequences include asthma, type 1 diabetes, low-grade systemic inflammation, sleep apnea, and musculoskeletal disorders, particularly those affecting the lower limbs and feet [2, 3].

Generally, overweight condition and obesity are defined as abnormal or excessive fat accumulation that poses human health risks [4]. Therefore, body fat measurement is considered an essential aspect of childhood obesity research [5, 6]. In large-scale population surveys and public health screenings, some anthropometric indices or variables are commonly used as surrogates for body fat [7, 8]. Body mass index (BMI) is a highly recommended and widely used tool to define childhood obesity [9, 10]. Waist circumference (WC) has been extensively investigated as an indicator of extreme body fat and health risks among children and adults [7]. WC and related variables, such as waist circumference-to-hip circumference ratio and waist-to-height ratio, are regarded as reliable factors corresponding to the level of visceral adipose tissues; these factors are associated with some risk factors of metabolic diseases among adults and children [11–14].

Compared with BMI, the WC of children provides a more reliable estimate of visceral adipose tissues measured with MRI at the level of the fourth lumbar vertebra (65% versus 56% variance) [15]. In multivariate regression models, WC is significantly more efficient than BMI in predicting health risk factors [16–18]. Therefore, WC measurements provide unique predictive information regarding health risks [8].

However, standardized protocols for WC measurement have not been established [19, 20]. The requirement for a standardized anatomic point of WC measurement, which is selected as the best predictor of adverse cardiometabolic outcomes on a risk-weighted basis, has been proposed in previous studies [21]. Four measurement sites are commonly used in studies and tests [22, 23]: above the iliac crest [24], at the midpoint between the lowest rib and the superior border of the iliac crest [25], below the lowest rib [22, 23], and at the narrowest waist [26]. In China, WC is measured 1 cm above the umbilicus [27].

The implications of measuring WC at different sites have been widely investigated in white and African American children. Wang et al. used four WC measurement sites in 111 subjects aged 7–83 years and observed that WC values at different sites are various, highly repeatable, and correlated with body fat in the trunk and total body in a gender-dependent manner [22]. Three WC measurements are correlated with BMI standard deviation scores among children, but bias and variability are observed in such measurements [28]. WC measurements are also strongly correlated with abdominal fat and cardiometabolic risks [23, 29–31]. WC measurements at different sites are similarly correlated with cardiometabolic risks or abdominal fat among children [23, 29, 30]. Among measurement sites, the narrowest waist is the best predictor of cardiometabolic risks in Brazilian children [31]. The narrowest waist and the midpoint between the lowest rib and the superior border of the iliac crest may indicate the WC measurements most closely associated with cardiometabolic risks among overweight children in Canada [32].

Most of the published studies have focused on white and African American children. The body fat content of Asian children and adolescents is higher than that of African-descent or white children and adolescents within the same BMI level [33–35]. The degree of central fat deposition in Chinese is also greater than that in whites and blacks [36, 37]. However, the differences of WC measurements at different anatomical sites among Chinese children have been rarely explored. Such differences may affect the relationship between WC and total body fat or trunk body fat.

This study aimed to compare WC measurements at five anatomical sites and to investigate the association between WC measurements and body composition in Chinese children.

## 2. Materials and Methods

### 2.1. Participants

A total of 255 Chinese children and adolescents aged 9–19 years were recruited from schools in Shanghai, China. Of this number, 127 were males and 128 were females. A stratified sampling method was used to recruit a heterogeneous sample covering a wide range of body compositions and ages on the basis of age- and gender-specific BMI distributions among Chinese children [38]. Written informed consent was obtained from the participants and their parents, and the study was approved by the Shanghai University of Sport Ethics Committee.

### 2.2. Anthropometric Measurements

A trained investigator measured the following parameters from the participants who wore minimal clothing and were barefoot. Body weight was measured to the nearest 0.1 kg using a standard scale (Tanita 543, Tanita, Japan). Body height was determined to the nearest 5 mm using a standard stadiometer (Holtain, UK). BMI was calculated as body weight (kg)/body height (m^2^).

WC was measured to the nearest 1 mm at five anatomical sites and recorded at minimal respiration using an inelastic measuring tape while the participants stood and maintained their balance on both feet with their arms hanging freely. The whole measurement was also completed by a well-trained investigator. Each measurement was repeated twice; if the measured values were within 0.5 cm of one another, then their average was calculated. If the difference between the two measurements exceeded 0.5 cm, then a third measurement was conducted. The five WC measurement points were (1) immediately below the lowest rib (WC1), (2) the narrowest part of the torso (WC2), (3) the midpoint between the lowest rib and the superior border of the iliac crest (WC3), (4) 1 cm above the umbilicus (WC4), and (5) immediately above the superior border of the iliac crest (WC5).

### 2.3. Body Composition Measurements

Dual-energy X-ray absorptiometry (GE Lunar Prodigy, GE Healthcare, USA) was used to measure body composition. In a supine position, each participant was scanned in fast mode. This procedure was performed for at least 5 min or was completed depending on the body height of the participant. FM, % BF, FM in the trunk, and % BF in the trunk were subjected to data analysis. All of the dual-energy X-ray absorptiometry scan measurements were conducted by a trained investigator. DEXA is the most suitable method to measure body fat in the general population. This method measures adipose tissue with great accuracy, safe low radiation, and short scanning time [39].

### 2.4. Statistical Analysis

Data were represented as mean and standard deviation (SD). The 5th and 95th percentiles of the physical characteristics of participants were also reported. Statistical analysis was conducted using SPSS 17.0 (SPSS Inc., Chicago, USA).

Data were examined to determine the normal distribution via Kolmogorov-Smirnov test. Nonnormally distributed data were transformed into normalized data through log transformation. Gender differences in anthropometric measurements and body compositions were identified via independent *t*-test.

Comparisons among the mean WC measurements were performed through ANOVA with repeated measures, and Bonferroni adjustment was conducted for multiple comparisons. After adjusting for age, we assessed the relationship between anthropometric measurements (BMI and WC measurements), FM, % BF, FM in the trunk, and % BF in the trunk by determining partial correlation coefficients.

In all of these tests, males and females were analyzed separately. Significance was set at *p* < 0.05.

## 3. Results

The characteristics of the participants are presented in Table 1. No gender differences were observed in terms of age, FM, and FM in the trunk. The height, weight, and WCs of the females were lower than those of the males. By comparison, % BF and % BF in the trunk of the females were higher than those of the males (Table 1).

In males, the value for each measurement site was significantly different from all other individual sites, with WC2 < WC1 < WC3 < WC4 < WC5 (Table 2). Similar results, except WC3 and WC4, were observed in females. The differences in WC magnitudes were more obvious in females than in males (Table 2).

All of the WC measurements were very strongly correlated with one another in both genders. For both genders, all the WC measured sites were very strongly correlated with BMI (Table 3).

Table 4 shows the partial correlations of BMI and WC at each site with FM, % BF, FM in the trunk, and % BF in the trunk. For both genders, BMI was very strongly correlated with FM (*p* < 0.001; for males, *r* = 0.925; for females, *r* = 0.927), % BF (*p* < 0.001; for males, *r* = 0.836; for females, *r* = 0.824), FM in the trunk (*p* < 0.001; for males, *r* = 0.923; for females, *r* = 0.908), and % BF in the trunk (*p* < 0.001; for males, *r* = 0.848; for females, *r* = 0.826). In males, all WC measurements had very strong correlations with FM (*r* = 0.904 to 0.927, *p* < 0.001), % BF (*r* = 0.800 to 0.833, *p* < 0.001), FM in the trunk (*r* = 0.914 to 0.934, *p* < 0.001), and % BF in the trunk (*r* = 0.822 to 0.851, *p* < 0.001). In females, the measured WCs were very strongly correlated with FM (*r* = 0.881 to 0.895, *p* < 0.001) and FM in the trunk (*r* = 0.882 to 0.891, *p* < 0.001). The age-adjusted correlations between WC measurements and % BF for females were 0.769 to 0.783 (*p* < 0.001). By comparison, such correlations between WC measurements and % BF in the trunk of females were 0.791 to 0.807 (*p* < 0.001) (Table 4).

## 4. Discussion

This study is the first to evaluate the relationship between WC measurements and body composition in Chinese children. The relatively large sample with a large age range is a marked strength of the current study. We identified significant differences in absolute WC values among Chinese children, except for the WC measured at the 1 cm above the umbilicus and the midpoint between the lowest rib and the iliac crest in females. The correlations between the WC measurements at five sites and FM, % BF, FM in the trunk, and % BF in the trunk were similarly high in both genders.

Similar to other studies on children [22, 23, 29, 30, 32], our study demonstrated that the absolute WC measurements varied among the five anatomical sites of male and female Chinese children. Wang et al. reported that the various WC measurements obtained at four commonly used sites, namely, the midpoint between the lowest rib and the iliac crest, iliac crest, narrowest waist, and immediately below the rib, differ in absolute values depending on gender [22]. Johnson et al. measured WC at four sites (narrowest waist, the midpoint between the lowest rib and the iliac crest, iliac crest, and umbilicus) in overweight children; the study also found differences that exist in the four WC measurement sites [32]. Harrington et al. had similar findings with the study of Johnson et al. Conversely, the absolute values of averaged WC significantly differ in gender-by-race groups [30]. These findings suggested that WC differs based on the measurement sites, and the different WC measurements are not interchangeable. With extensive investigations on WC as an indicator of obesity-related health risks and overweight in children and adolescents, many WC percentiles for children have been developed in different countries, including USA [40], Canada [41], the United Kingdom [42], Australia [43], Italy [21], China, and Hong Kong [44, 45]. In the development of WC percentiles in various countries, the circumference is measured at the different sites, including the iliac crest [41, 46], narrowest waist [42], umbilicus [43, 47], and at the midpoint between the lowest rib and the iliac crest [45, 48–51]. However, this varying measurement may lead to misinterpretation when these references are considered for comparisons between studies involving different measurement protocols, weight status management, and overweight and obesity prevalence, because the extent of changes may vary as a function of the WC site. Our findings suggested that WCs obtained at the five positions were not comparable to the absolute values among Chinese children, except for the WC measured at 1 cm above the umbilicus and the WC obtained at the midpoint between the lowest rib and the iliac crest in females. Our results support the proposition that standardizing the anatomic point for WC measurements is required to observe human growth over time or perform a comparison across countries.

Our findings also demonstrated that the WC of each measurement site was significantly different from those observed in other individual sites in males: WC2 (the narrowest) < WC1 (the lowest rib) < WC3 (the midpoint between the iliac crest and the lowest rib) < WC4 (1 cm over the umbilicus) < WC5 (iliac crest). Similar results were found in females, except WCs measured at the midpoint between the iliac crest and the lowest rib and those obtained at 1 cm above the umbilicus. The WC in the narrowest site is significantly smaller than that at the iliac crest [22, 32]. Different WCs are also found at the lowest rib, the midpoint between the iliac crest and the lowest rib, and the iliac crest [23, 29]. Our results are consistent with those observed in the previous studies. Harrington et al. investigated the differences in WCs in terms of race and gender and found that the WC detected at the narrowest waist is significantly smaller than those at the midpoint between the iliac crest and the lowest rib, the umbilicus, and the iliac crest [30]. They also found that the WCs at the midpoint between the iliac crest and the lowest rib do not significantly differ from those at the umbilicus and the iliac crest in African American males [30]. For white males and African American females, the WCs at the iliac crest do not significantly differ from those at the umbilicus and also yield higher values than those at the narrowest and the midpoint between the iliac crest and the lowest rib [30]. For white females, the WC measurements were significantly different from one another [30]. These findings on white and African American females are inconsistent with those observed in Chinese children possibly because of ethnic differences in body compositions [33–35]. In the present study, the differences in WC magnitudes were more obvious in females than in males. This result is consistent with the findings detected in a previous study [23]. Consistent with previous results [22, 23, 28], our findings revealed that the five WC measurements were highly correlated with on another but were different in their magnitude. Therefore, the WCs measured at different sites could not be used interchangeably in Chinese children. This phenomenon was more obvious in females than in males probably because of gender differences in body shape.

This study to determine the correlation between WC measurements and body fat can contribute to developing the best WC measurement for predicting health-related risk factors. In our study, all the WC measurements were highly correlated with BMI, although bias was detected among the WC measurements. Our findings confirmed the results described in previous studies [23, 28]. In our study, the WC measurements were highly correlated with FM, % BF, FM in the trunk, and % BF in the trunk in male and female Chinese children. In a previous study, WC measurements at four sites were similarly correlated with FM, % BF in the trunk, and FM in the trunk in both genders but correlated significantly with % BF in females only [22]. Compared with WC measurements at the three other measurement sites, WC measured above the iliac crest is closely associated with body fat mass [22]. The WC measured above the iliac crest is also more strongly correlated with % BF than the WC determined 4 cm above the umbilicus in white children [22]. In our study, WC measurements were more strongly correlated with BMI and body composition variables in males than in females (Table 4). This finding may be attributed to gender differences in terms of body shape. In our study, the five WC measurements exhibited a comparable association with BMI, FM, % BF, FM in the trunk, and % BF in the trunk in males and females. This finding differed from the findings in previous studies. One of the most important reasons is the ethnic difference in body composition. Body fat distribution and body composition differ across racial or ethnic groups in adults and children [34, 36]. At same BMI level, the comparative data on Asians and Western populations reveal that Asians yield a higher % BF, and blacks and whites have less % BF [52, 53]. Asians exhibit a lower appendicular skeletal muscle mass, lower gynoid fat, and longer trunk lengths than Caucasians [54]. Asians also generally possess a higher abdominal fat than Caucasians [55]. Thus, the characteristics of fat distribution in Chinese may explain our findings that the WCs measured at different sites are significantly correlated with the % BF and % BF in the trunk of Chinese children.

Despite a number of strengths, our study has several limitations. One of the limitations of the present study is that, in our assessment of the relationship between WC and body fat distribution, the stage of sexual maturation is not considered, and this factor may influence the relationship. This study is cross-sectional, and future studies should consider using a longitudinal design to evaluate the best WC measurement to determine the health risk in Chinese children. WC reflects abdominal fat issue and cannot differentiate between visceral and subcutaneous fat storage. Previous studies suggested that WC should be an index of visceral obesity. In the current study, visceral fat and cardiometabolic risk factors were not measured, which is another limitation of our study. The relationships between WC with visceral adipose tissues and cardiometabolic risk factors should be extensively examined in future studies.

## 5. Conclusions

This study revealed that the WCs measured at the five commonly used sites in Chinese children are different. The WCs are also correlated significantly with FM, % BF, FM in the trunk, and % BF in the trunk. Therefore, a standardized anatomic point is required for WC measurement based on the relationship between WC measurements at different sites and body fat.

## Figures and Tables

**Table 1:** Descriptive statistics of physical characteristics of participants.

	Male (*n* = 127)			Female (*n* = 128)			*p* value	95% CI of mean difference
	Mean (SD)	5th percentiles	95th percentiles	Mean (SD)	5th percentiles	95th percentiles		
Age (y)	13.81 (2.85)	9.10	18.46	13.66 (2.85)	9.09	18.57	0.670	−0.55–0.85
Height (cm)	161.80 (14.04)	135.90	181.00	154.31 (9.77)	136.79	167.91	<0.001	4.51–10.47
Weight (kg)	55.9 (17.0)	33.80	92.58	47.4 (11.3)	30.80	65.71	<0.001	4.90–12.02
BMI (kg/m^2^)	20.96 (4.16)	16.03	30.02	19.69 (3.25)	15.20	25.22	0.008	0.34–2.18
WC1 (cm)	69.52 (10.32)	56.41	93.02	62.48 (7.00)	52.02	75.08	<0.001	4.87–9.21
WC2 (cm)	69.01 (10.55)	55.76	91.64	62.01 (6.93)	51.60	73.96	<0.001	4.80–9.20
WC3 (cm)	70.06 (11.25)	56.33	93.35	63.86 (7.51)	52.80	76.82	<0.001	3.83–8.55
WC4 (cm)	70.77 (11.73)	56.53	95.41	63.80 (7.52)	53.06	76.83	<0.001	4.45–9.30
WC5 (cm)	73.09 (11.78)	58.20	96.67	67.68 (8.32)	55.23	80.27	<0.001	2.90–7.93
% BF (%)	20.77 (9.90)	7.94	38.64	27.85 (7.67)	14.99	40.70	<0.001	−9.26–−4.90
FM (kg)	12.06 (9.02)	3.52	31.90	13.49 (6.42)	5.07	24.17	0.147	−3.35–0.50
% BF in trunk (%)	20.77 (10.91)	7.14	42.60	27.28 (8.66)	13.25	41.72	<0.001	−8.94–−4.08
FM in trunk (kg)	5.66 (4.89)	1.42	17.65	6.18 (3.29)	1.91	11.89	0.320	−1.55–0.51

WC1: immediately below the lowest rib; WC2: at the narrowest part of the torso, above the umbilicus, and below the xiphoid process; WC3: midpoint between the lowest rib and the iliac crest; WC4: 1 cm above the umbilicus; WC5: immediately above the superior border of the iliac crest.

% BF: body fat percentage; FM: fat mass; % BF in trunk: body fat percentage in the trunk; FM in trunk: fat mass in the trunk.

**Table 2:** Comparison of 5 waist circumference sites and gender differences (mean (SD, 95% CI of mean difference)).

	Comparison of 5 waist circumference sites		Comparison on difference of 5 WC sites between genders	
	Females (*n* = 128)	Males (*n* = 127)	*p* value	95% CI of mean difference
WC5 versus WC1 (cm)	5.19 (2.55, 4.55–5.83)^*∗*^	3.57 (3.01, 2.80–4.33)^*∗*^	<0.001	0.94–2.31
WC5 versus WC2 (cm)	5.66 (2.56, 5.01–6.30)^*∗*^	4.07 (2.81, 3.36–4.79)^*∗*^	<0.001	0.92–2.25
WC5 versus WC3 (cm)	3.81 (1.91, 3.33–4.29)^*∗*^	3.03 (2.39, 2.42–3.63)^*∗*^	0.004	0.25–1.32
WC5 versus WC4 (cm)	3.88 (2.13, 3.34–4.41)^*∗*^	2.41 (2.21, 1.85–2.97)^*∗*^	<0.001	0.93–2.00
WC4 versus WC1 (cm)	1.32 (1.45, 0.95–1.68)^*∗*^	1.15 (2.30, 0.57–1.74)^*∗*^	0.497	−0.31–0.64
WC4 versus WC2 (cm)	1.78 (1.39, 1.43–2.13)^*∗*^	1.66 (1.94, 1.17–2.15)^*∗*^	0.568	−0.30–0.54
WC4 versus WC3 (cm)	−0.07 (1.35, −0.41–0.27)	0.61 (1.41, 0.26–0.97)^*∗*^	<0.001	−1.02–−0.34
WC3 versus WC1 (cm)	1.38 (1.44, 1.02–1.75)^*∗*^	0.54 (1.83, 0.07–1.00)^*∗*^	<0.001	0.44–1.25
WC3 versus WC2 (cm)	1.85 (1.39, 1.50–2.20)^*∗*^	1.05 (1.48, 0.67–1.42)^*∗*^	<0.001	0.45–1.15
WC1 versus WC2 (cm)	0.47 (0.72, 0.29–0.65)^*∗*^	0.51 (0.88, 0.29–0.73)^*∗*^	0.665	−0.24–0.15

WC1: immediately below the lowest rib; WC2: at the narrowest part of the torso, above the umbilicus, and below the xiphoid process; WC3: midpoint between the lowest rib and the iliac crest; WC4: 1 cm above the umbilicus; WC5: immediately above the superior border of the iliac crest.

^*∗*^*p* < 0.001, significant difference between WC measurement sites within each gender.

**Table 3:** Age-controlled correlations between BMI and waist circumferences measured at 5 sites in males (*∗*) and females (*∗∗*).

	BMI	WC1	WC2	WC3	WC4	WC5
BMI	—^*∗*^	0.951^*∗*^	0.952^*∗*^	0.952^*∗*^	0.950^*∗*^	0.939^*∗*^
WC1	0.889^*∗∗*^	—^*∗*^	0.996^*∗*^	0.989^*∗*^	0.986^*∗*^	0.966^*∗*^
WC2	0.883^*∗∗*^	0.994^*∗∗*^	—^*∗*^	0.992^*∗*^	0.990^*∗*^	0.970^*∗*^
WC3	0.887^*∗∗*^	0.980^*∗∗*^	0.982^*∗∗*^	—^*∗*^	0.992^*∗*^	0.977^*∗*^
WC4	0.879^*∗∗*^	0.980^*∗∗*^	0.982^*∗∗*^	0.981^*∗∗*^	—^*∗*^	0.980^*∗*^
WC5	0/879^*∗∗*^	0.951^*∗∗*^	0.953^*∗∗*^	0.973^*∗∗*^	0.964^*∗∗*^	—^*∗*^

BMI: body mass index; WC1: immediately below the lowest rib; WC2: at the narrowest part of the torso, above the umbilicus, and below the xiphoid process; WC3: midpoint between the lowest rib and the iliac crest; WC4: 1 cm above the umbilicus; WC5: immediately above the superior border of the iliac crest.

All correlation significant at *p* < 0.001.

**Table 4:** Age-controlled correlations relating BMI, waist circumference at each site to fat mass, body fat percentage, fat mass in trunk, and fat percentage in trunk for each gender.

	Male (*n* = 127)				Female (*n* = 128)			
	FM	% BF	FM in trunk	% BF in trunk	FM	% BF	FM in trunk	% BF in trunk
WC1	0.904	0.800	0.914	0.822	0.885	0.769	0.884	0.792
WC2	0,913	0.812	0.923	0.832	0.881	0.767	0.882	0.791
WC3	0.918	0.826	0.929	0.846	0.892	0.782	0.888	0.804
WC4	0.927	0.833	0.934	0.851	0.889	0.777	0.884	0.798
WC5	0.919	0.822	0.925	0.840	0.895	0.783	0.891	0.807

WC1: immediately below the lowest rib; WC2: at the narrowest part of the torso, above the umbilicus, and below the xiphoid process; WC3: midpoint between the lowest rib and the iliac crest; WC4: 1 cm above the umbilicus; WC5: immediately above the superior border of the iliac crest.

% BF: body fat percentage; FM: fat mass; % BF in trunk: body fat percentage in the trunk; FM in trunk: fat mass in the trunk

All correlation significant at *p* < 0.001.
